# Brain-inspired multimodal motion and fine-grained action recognition

**DOI:** 10.3389/fnbot.2024.1502071

**Published:** 2025-01-24

**Authors:** Yuening Li, Xiuhua Yang, Changkui Chen

**Affiliations:** ^1^Wuhan Sports University, Wuhan, China; ^2^School of Physical Education and Training, Party School of Shandong Provincial Committee of the Communist Party of China (Shandong Administrative Institute), Jinan, Shandong, China

**Keywords:** brain-inspired, multimodal, action recognition, CLIP, clustering algorithms

## Abstract

**Introduction:**

Traditional action recognition methods predominantly rely on a single modality, such as vision or motion, which presents significant limitations when dealing with fine-grained action recognition. These methods struggle particularly with video data containing complex combinations of actions and subtle motion variations.

**Methods:**

Typically, they depend on handcrafted feature extractors or simple convolutional neural network (CNN) architectures, which makes effective multimodal fusion challenging. This study introduces a novel architecture called FGM-CLIP (Fine-Grained Motion CLIP) to enhance fine-grained action recognition. FGM-CLIP leverages the powerful capabilities of Contrastive Language-Image Pretraining (CLIP), integrating a fine-grained motion encoder and a multimodal fusion layer to achieve precise end-to-end action recognition. By jointly optimizing visual and motion features, the model captures subtle action variations, resulting in higher classification accuracy in complex video data.

**Results and discussion:**

Experimental results demonstrate that FGM-CLIP significantly outperforms existing methods on multiple fine-grained action recognition datasets. Its multimodal fusion strategy notably improves the model's robustness and accuracy, particularly for videos with intricate action patterns.

## 1 Introduction

In the context of rapid advancements in information technology, motion action recognition has emerged as a pivotal research area within computer vision and artificial intelligence (Kong and Fu, 2022). This technology is receiving heightened attention due to its critical role in enhancing the accuracy and intelligence of applications such as intelligent surveillance systems, medical rehabilitation, sports analysis, and human-computer interaction (Sun et al., 2022). The ability to automatically recognize and analyze human actions enables significant labor cost reductions and improves the speed and precision of system responses, thus contributing to smarter and more efficient solutions. Motion action recognition research, therefore, is not only a natural progression in technological innovation but also addresses pressing real-world application challenges (Elharrouss et al., 2021).

Traditional approaches to action recognition, including symbolic AI and knowledge-based methods, rely heavily on predefined rules and logical reasoning. These methods offer certain advantages, such as low computational requirements and straightforward implementation, and perform adequately in structured environments with well-defined rules. For example, momentum-based methods analyze motion changes by computing object speed and direction, making them useful for video surveillance and motion tracking (Rao et al., 2021). Gradient-based methods, which analyze brightness and color changes within video frames, can capture subtle motion nuances effectively (Xiao et al., 2022), while logistic regression provides a simple statistical approach to classify basic actions (Sun et al., 2022). Despite these advantages, such traditional methods are sensitive to noise and struggle to capture complex, nonlinear motion patterns, limiting their applicability in dynamic environments with large-scale, diverse data.

In recent years, machine learning-based algorithms have gained popularity for motion action recognition as they allow for automatic feature extraction from large datasets, improving both generalization and recognition accuracy. Methods such as Principal Component Analysis (PCA) are used for dimensionality reduction, extracting representative features to enhance efficiency and accuracy (Shiripova et al., 2020). Ensemble models like Random Forests employ multiple decision trees with a voting mechanism, enhancing stability and resilience against noise (Langroodi et al., 2021). Multi-Layer Perceptrons (MLPs) utilize nonlinear mapping across multiple layers, effectively recognizing complex actions through higher classification precision. However, these methods require substantial computational resources and heavily rely on large annotated datasets, which pose challenges in practical, resource-constrained applications.

To handle the limitations of statistical and machine learning techniques with high-dimensional time-series data, deep learning-based algorithms have become central to motion action recognition. These methods achieve superior recognition by learning hierarchical features from data, handling multimodal inputs, and managing complex temporal dependencies. Convolutional Neural Networks (CNNs) excel at learning spatial features in video frames and capturing local motion features (Chen et al., 2021). Recurrent Neural Networks (RNNs) and Long Short-Term Memory (LSTM) networks are effective in handling temporal dynamics by incorporating memory units for long-term dependencies (Majd and Safabakhsh, 2020). More recently, attention mechanisms in Transformer models have significantly improved action recognition, weighting key features to handle intricate action sequences (Liu et al., 2022). Although these methods excel in processing large-scale, complex datasets, they demand substantial computational resources, complex training processes, and access to extensive labeled data.

Existing deep learning methods face significant challenges in fine-grained action recognition, particularly in accurately capturing subtle and intricate actions within multimodal data. Traditional models often struggle with the complexities of fusing multimodal information and lack the sensitivity to nuanced motion variations. To address these limitations, we propose a novel architecture, FGM-CLIP (Fine-Grained Motion CLIP), specifically designed to enhance the recognition of detailed actions within a multimodal framework. FGM-CLIP leverages the robust joint representation learning capabilities of Contrastive Language-Image Pretraining (CLIP) and extends this to video action recognition, capturing fine motion details through an end-to-end architecture. This innovative approach combines a CLIP-based feature extraction module, a fine-grained motion encoder, and a multimodal fusion layer to integrate both visual and motion features seamlessly. By jointly optimizing these features, FGM-CLIP achieves high precision in classifying complex actions, thus pushing the boundaries of fine-grained action recognition. Through this architecture, we aim to provide a more effective tool for detailed action analysis and set a foundation for future multimodal learning advancements.

- FGM-CLIP introduces a CLIP-based feature extraction module and combines it with a fine-grained action encoder to innovatively enhance the capability to capture complex action details.
- This method demonstrates efficiency and versatility across multiple scenarios, accurately identifying fine-grained actions in various video datasets with strong adaptability.
- Experimental results indicate that FGM-CLIP significantly improves classification accuracy across several action recognition benchmark tests, showing exceptional performance particularly in fine-grained action classification tasks.

## 2 Related work

### 2.1 Action recognition

Action recognition has emerged as a crucial area of research within computer vision, primarily fueled by the exponential growth of video data across various platforms. Early techniques for action recognition were predominantly based on manually designed feature extraction methods, such as optical flow and trajectory-based approaches (Li Q. et al., 2024). While these methods were somewhat effective for simpler action scenarios, they faced significant challenges when dealing with complex and nuanced action sequences, where subtle differences and contextual information are vital for accurate classification. The introduction of deep learning has brought about a transformative shift in action recognition, with Convolutional Neural Networks (CNNs) and Recurrent Neural Networks (RNNs) becoming prominent tools in this domain. CNNs are adept at extracting rich spatial features from individual video frames, capturing essential visual elements that characterize actions. Meanwhile, RNNs, particularly Long Short-Term Memory (LSTM) networks, are designed to model temporal sequences, enabling the capture of dynamic aspects inherent in video content (Wang et al., 2019b). The integration of spatial and temporal data through these architectures has led to significant improvements in action recognition accuracy. Despite these advancements, traditional deep learning methods still encounter limitations when processing long sequences, handling complex backgrounds, and discerning subtle differences in actions. To address these challenges, researchers have turned to multimodal learning techniques that integrate various modalities–such as visual, motion, audio, and textual information–enhancing model performance by leveraging complementary data sources (Wang et al., 2019b). This approach allows for a more holistic understanding of actions, improving recognition capabilities in diverse contexts. Additionally, the rise of Transformer-based models in action recognition has opened new avenues for exploration. Transformers excel in capturing long-range dependencies through self-attention mechanisms, allowing models to focus on relevant parts of the input sequence while considering the overall context (Li M. et al., 2024). This capability is particularly beneficial for recognizing complex actions that unfold over extended periods, as it enables the model to retain critical information from earlier frames that may influence later actions. Overall, the ongoing evolution in action recognition methodologies, fueled by deep learning and multimodal integration, is paving the way for more accurate and robust systems capable of understanding intricate human activities in dynamic environments.

### 2.2 Action recognition with CLIP models

Contrastive Language-Image Pre-training (CLIP) models have shown remarkable potential in bridging the gap between visual and textual data, creating unified feature representations that facilitate zero-shot learning and robust cross-modal understanding (Lin et al., 2022). In the context of action recognition, recent studies have adapted CLIP's joint embedding capabilities to video tasks, aiming to leverage both visual cues from frames and semantic cues from textual descriptions (Fishel and Loeb, 2012). However, applying CLIP to action recognition introduces unique challenges. First, video data encompasses complex temporal dependencies that are not naturally suited to the static image-text pairs on which CLIP was originally trained (Wang et al., 2019a). Researchers have attempted to address this by fine-tuning CLIP for video action recognition or integrating it with temporal models, such as LSTMs and Transformers, to better capture the sequential nature of actions. Despite these efforts, capturing fine-grained motion details and ensuring temporal alignment between frames remain open challenges. Furthermore, CLIP's sensitivity to nuanced action variations is limited, which can impact its performance on fine-grained action recognition tasks where subtle differences are crucial (Liu et al., 2025).

### 2.3 Challenges in end-to-end learning for action recognition

End-to-end learning has emerged as a powerful approach in action recognition, offering the advantage of optimizing all components of the model simultaneously for a cohesive representation of both visual and motion cues. Yet, this approach is not without limitations (Sverrisson et al., 2021). For instance, directly applying end-to-end learning to multimodal data often results in inefficiencies in capturing the distinct dynamics of each modality. In action recognition, temporal dynamics play a critical role, requiring architectures that can manage not only visual feature extraction but also the temporal evolution of those features across frames (Wang et al., 2024). Traditional end-to-end models, such as CNN-LSTM or CNN-Transformer hybrids, often struggle to achieve this balance effectively, especially in the context of complex, fine-grained actions (Wang et al., 2016). Furthermore, the integration of domain-specific priors, such as known motion patterns or contextual information from the scene, into an end-to-end framework remains a challenging area. Without such priors, end-to-end models can overfit to spurious features in training data, limiting their ability to generalize to new environments or action types. To address these issues, recent approaches have incorporated multimodal fusion layers, domain adaptation techniques, and regularization methods to enhance model robustness and adaptability in diverse action recognition scenarios.

## 3 Methodology

### 3.1 Overview of our network

In this work, we introduce a novel architecture named FGM-CLIP (Fine-Grained Motion CLIP), specifically designed to enhance the recognition of fine-grained actions within a multimodal framework. The architecture effectively leverages the capabilities of Contrastive Language-Image Pre-training (CLIP) and integrates it with a fine-tuned motion recognition mechanism, addressing the prevalent challenges associated with detailed motion analysis in video data. By operating in an end-to-end manner, FGM-CLIP allows for the joint optimization of visual and motion features, enabling the model to capture subtle variations in actions that are critical for accurate recognition. The architecture comprises three main components: a CLIP-based feature extraction module, a fine-grained motion encoder, and a multimodal fusion layer. The CLIP-based feature extraction module utilizes the powerful capabilities of CLIP, which has been trained on a large corpus of image-text pairs. This module extracts rich visual features from the input video frames while simultaneously generating contextual textual representations. The ability of CLIP to understand both visual and linguistic information significantly enhances the model's performance, allowing it to leverage the semantic richness of textual descriptions during action recognition.

**Figure 1:**
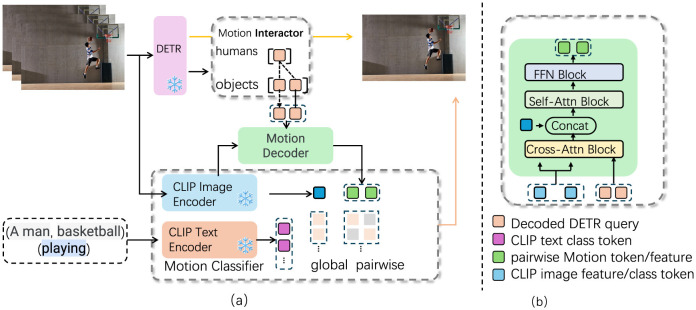
FGM-CLIP architecture illustration, showing the detailed processing flow for fine-grained action recognition. **(a)** The system begins with the DETR model identifying objects and humans in video frames, followed by motion feature extraction using the Motion Interactor and Motion Decoder. CLIP's Image and Text Encoders generate both visual and textual representations, which are then processed by the Motion Classifier to produce global and pairwise motion tokens. **(b)** The Motion Decoder integrates these features using cross-attention, self-attention, and feed-forward blocks, resulting in an enriched representation for precise action classification.

Following feature extraction, the fine-grained motion encoder processes the motion data extracted from the video. This component is specifically designed to capture intricate details of the motion sequences, enabling the model to analyze temporal dynamics effectively. By focusing on the temporal aspect of the actions, the motion encoder ensures that even the slightest variations in movement are accounted for, which is essential when differentiating between actions that may appear visually similar but differ in subtle temporal dynamics. The final component, the multimodal fusion layer, synergizes the extracted visual and motion features. This layer combines information from both modalities, allowing the model to take advantage of the complementary strengths of visual and motion data. The fusion of these features enhances the model's ability to make precise action classifications, as it can consider both the visual appearance and the motion dynamics of the actions simultaneously. The primary innovation of our approach lies in the seamless integration of CLIP with motion-based analysis, which facilitates a comprehensive understanding of fine-grained actions. This integration not only harnesses the extensive visual and textual knowledge encoded in CLIP but also enables fine-tuning on the specific nuances of motion data. Consequently, FGM-CLIP is particularly relevant for tasks requiring the differentiation of actions that share visual similarities yet exhibit distinct temporal characteristics (as shown in Figure 1).

In our model, the text encoder is designed to process descriptive textual data related to each action, rather than just simple action labels. Specifically, “text” refers to carefully crafted descriptions that capture the context and finer details of each action. For instance, instead of using a basic label like “running,” the text input may consist of phrases such as “an athlete running on a track” or “a person sprinting across a field,” which provide richer semantic information that aligns closely with the action being performed. These descriptions are derived from existing datasets or manually created to align with the nuances of each action class, ensuring that the text captures detailed aspects of the motion and context. The text encoder, based on CLIP's pretrained language model, encodes these descriptive texts into a feature space that aligns with the visual embeddings generated by the image encoder. During training, we leverage CLIP's multimodal embedding space to map both visual and textual representations onto a shared space, enabling the model to associate nuanced textual descriptions with corresponding visual cues. This integration allows the model to utilize semantic information from the text to enhance its ability to distinguish fine-grained actions that may look visually similar but differ in context or subtle motion characteristics.

In the following sections, we describe the architecture and components of FGM-CLIP in detail. In Section 3.2, we introduce the mathematical formulation of the problem, outlining the key challenges and objectives. Section 3.3 details the novel architecture of FGM-CLIP, emphasizing the design choices that enable fine-grained action recognition. Finally, in Section 3.4, we discuss the strategies employed to integrate domain-specific priors into the model, enhancing its ability to generalize across diverse datasets.

### 3.2 Preliminaries

The task of fine-grained motion action recognition involves identifying and classifying subtle and often nuanced movements within video sequences. Formally, let $V=\left\{v_{1},v_{2},\ldots ,v_{N}\right\}$ represent a set of *N* video clips, where each video clip *v*_*i*_ consists of a sequence of frames {*f*_*i*1_, *f*_*i*2_, …, *f*_*iT*_}, and *T* denotes the number of frames in the clip. Each video clip *v*_*i*_ is associated with a ground-truth label $y_{i}\in C$, where C is the set of possible action categories. Given this setup, the objective is to learn a function F:V→C that maps each video clip *v*_*i*_ to its corresponding action label *y*_*i*_. This mapping function F is parameterized by a deep neural network, specifically designed to handle the multimodal nature of video data, which includes both visual features and temporal motion cues. To achieve this, we utilize a contrastive learning-based approach, inspired by the CLIP model, where a large-scale pre-trained network is fine-tuned on the target dataset for action recognition. The network F can be decomposed into three main components: a visual encoder $E_{v}$, a motion encoder $E_{m}$, and a fusion module G. The visual encoder $E_{v}$ extracts spatial features from individual frames, while the motion encoder $E_{m}$ captures temporal dynamics from the sequence of frames. The fusion module G combines these features to generate a final representation, which is then used for classification. The learning process involves minimizing a loss function L that captures the discrepancy between the predicted labels and the ground-truth labels. Specifically, we define the loss as follows:


(1) $$\begin{array}{l} L\left(\theta \right)=-\frac{1}{N}\overset{N}{\underset{i=1}{\sum }}\log p\left(y_{i}|v_{i};\theta \right) \end{array}$$

where θ denotes the parameters of the network F, and *p*(*y*_*i*_|*v*_*i*_; θ) is the probability assigned to the correct label *y*_*i*_ by the model.

In addition to this classification loss, we incorporate a contrastive loss to align the visual and motion representations. Let $V_{t}$ and $M_{t}$ denote the visual and motion feature spaces, respectively, at time step *t*. The contrastive loss is defined as:


(2) $$\begin{array}{l} L_{\text{contrastive}}\left(\theta \right)=\frac{1}{T}\overset{T}{\underset{t=1}{\sum }}\left(||E_{v}\left(f_{it};\theta_{v}\right)-E_{m}\left(f_{it};\theta_{m}\right)|{|}^{2}\right) \end{array}$$

where θ_*v*_ and θ_*m*_ represent the parameters of the visual and motion encoders, respectively. This loss ensures that the visual and motion features are closely aligned, enabling the network to effectively capture the fine-grained nuances of actions.

Finally, the overall loss function used to train the network is a weighted sum of the classification loss and the contrastive loss:


(3) $$\begin{array}{l} L_{\text{total}}\left(\theta \right)=L\left(\theta \right)+\lambda L_{\text{contrastive}}\left(\theta \right) \end{array}$$

where λ is a hyperparameter that balances the two loss components. The training process optimizes this total loss to learn the parameters θ that best distinguish between different fine-grained actions in the video data. This formulation sets the stage for the detailed exploration of the model architecture and strategies employed to achieve robust fine-grained action recognition, which are presented in the subsequent sections.

### 3.3 Motion-visual synergy module

**Figure 2:**
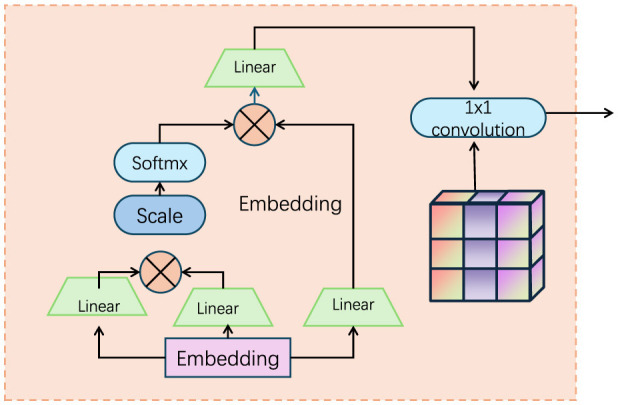
Diagram of the Motion-Visual Synergy Module (MVSM), illustrating the integration of spatial and temporal features extracted by visual and motion encoders. The module leverages embedding layers, scaling, and linear transformations to combine dynamic and static information, with a 1 × 1 convolution layer as the final step to produce comprehensive action representations.

To effectively capture and integrate the intricate dynamics of fine-grained actions, we introduce the Motion-Visual Synergy Module (MVSM) as the core component of the FGM-CLIP architecture. The MVSM is designed to synergize the spatial and temporal information extracted by the visual and motion encoders, respectively, ensuring that the final action representations are both comprehensive and discriminative (as shown in Figure 2).

#### 3.3.1 Feature extraction

In the first stage, we aim to extract meaningful features from both the visual and motion information contained in the video data. This process leverages two specialized encoders: the visual encoder $E_{v}$ for capturing spatial features from individual frames, and the motion encoder $E_{m}$ for extracting temporal features based on motion dynamics. The visual encoder $E_{v}$ processes each frame *f*_*it*_ from the video clip *v*_*i*_, producing a set of spatial features v_*it*_. These spatial features encapsulate the visual content present in each frame, such as object appearances and scene layouts. Mathematically, the spatial features are computed as follows:


(4) $$\begin{array}{l} {\text{v}}_{it}=E_{v}\left(f_{it};\theta_{v}\right) \end{array}$$

Here, *f*_*it*_ represents the *t*-th frame from video clip *v*_*i*_, and θ_*v*_ denotes the parameters of the visual encoder $E_{v}$, which is typically a deep convolutional neural network (CNN) pre-trained on large-scale image datasets.

Simultaneously, the motion encoder $E_{m}$ processes temporal information by analyzing the motion between consecutive frames. The motion information can be derived from optical flow or other motion cues that capture the pixel-wise displacement between two consecutive frames, *f*_*it*_ and *f*_*i*(*t*−1)_. The temporal features m_*it*_, which encode the dynamic aspects of the video, are computed as:


(5) $$\begin{array}{l} {\text{m}}_{it}=E_{m}\left(\text{OpticalFlow}\left(f_{it},f_{i\left(t-1\right)}\right);\theta_{m}\right) \end{array}$$

In this equation, OpticalFlow(*f*_*it*_, *f*_*i*(*t*−1)_) represents the optical flow calculated between the current frame *f*_*it*_ and the previous frame *f*_*i*(*t*−1)_, while θ_*m*_ denotes the parameters of the motion encoder $E_{m}$, which can be designed using CNNs or recurrent networks like LSTMs to capture the temporal dependencies in the video data. By combining the spatial features v_*it*_ and the temporal features m_*it*_, we obtain a comprehensive representation of both the static and dynamic elements in the video clip, which can then be used for subsequent stages of the video analysis task.

#### 3.3.2 Temporal alignment

In the second stage, we address the potential discrepancy in temporal resolution or synchronization between the visual and motion features. Since visual features v_*it*_ and motion features m_*it*_ may not be perfectly aligned in time, it is crucial to implement a temporal alignment mechanism to ensure that these features are synchronized. The goal is to adjust the motion features m_*it*_ such that they are temporally aligned with the corresponding visual features v_*it*_, resulting in an aligned motion feature set ${\tilde{\text{m}}}_{it}$. The temporal alignment is represented mathematically as:


(6) $$\begin{array}{l} {\tilde{\text{m}}}_{it}=\text{Align}\left({\text{m}}_{it},{\text{v}}_{it}\right) \end{array}$$

Here, ${\tilde{\text{m}}}_{it}$ denotes the aligned motion features for frame *t* in the video clip *v*_*i*_, and the function Align(·, ·) represents the alignment process. Several methods can be employed for this alignment:

Dynamic Time Warping (DTW): This technique computes the optimal alignment between two sequences (visual and motion features) by stretching or compressing one sequence in time to match the other. It minimizes the differences in temporal evolution between the features.


(7) $$\begin{array}{l} {\tilde{\text{m}}}_{it}=\text{DTW}\left({\text{m}}_{it},{\text{v}}_{it}\right) \end{array}$$

Attention-based Alignment: Another approach involves using attention mechanisms, which assign different weights to different temporal segments of the motion features m_*it*_ based on their relevance to the visual features v_*it*_. This allows the model to focus on temporally relevant parts of the motion sequence that correspond to visual cues.


(8) $$\begin{array}{l} {\tilde{\text{m}}}_{it}=\text{Attention}\left({\text{m}}_{it},{\text{v}}_{it}\right) \end{array}$$

The temporal alignment mechanism ensures that the temporal dynamics represented by the motion features are appropriately synchronized with the visual content, facilitating more effective joint processing in later stages of the model.

#### 3.3.3 Multimodal fusion

In the final stage, the aligned visual and motion features are fused to form a unified representation for each frame, capturing both spatial and temporal information. This fusion is critical for integrating the complementary aspects of the visual content (from the visual encoder) and motion dynamics (from the motion encoder). We utilize a multimodal fusion strategy that involves concatenating the aligned visual and motion features, followed by passing them through a fully connected layer. The fused feature representation for each frame *f*_*it*_ is given by:


(9) $$\begin{array}{l} {\text{h}}_{it}=\text{ReLU}\left({\text{W}}_{f}\left[{\text{v}}_{it};{\tilde{\text{m}}}_{it}\right]+{\text{b}}_{f}\right) \end{array}$$

In this equation: - W_*f*_ represents the weight matrix of the fully connected fusion layer, - b_*f*_ is the bias vector, - $\left[{\text{v}}_{it};{\tilde{\text{m}}}_{it}\right]$ denotes the concatenation of the spatial feature v_*it*_ and the aligned motion feature ${\tilde{\text{m}}}_{it}$, - ReLU(·) is the activation function that introduces non-linearity.

The output h_*it*_ is the fused feature vector for frame *f*_*it*_, encapsulating both the spatial and temporal attributes of the video at that particular time step. Once we have obtained fused features h_*it*_ for each frame, the next step is to aggregate these frame-level features to create a single video-level representation h_*i*_. This is achieved by averaging the fused features across all frames in the video:


(10) $$\begin{array}{l} {\text{h}}_{i}=\frac{1}{T}\overset{T}{\underset{t=1}{\sum }}{\text{h}}_{it} \end{array}$$

Here, *T* represents the total number of frames in the video, and h_*i*_ is the final aggregated video-level feature, which serves as a comprehensive representation of both the spatial content and temporal dynamics across the entire video. This video-level representation h_*i*_ is then passed to the classification layer for the final task, such as action recognition or video categorization.

### 3.4 Domain-specific prior integration

To improve the generalization of the FGM-CLIP model across different datasets and action categories, we incorporate domain-specific priors into the learning process. These priors, derived from existing knowledge about actions, motion patterns, and contextual information, allow the model to focus on relevant features and reduce overfitting to training data.

#### 3.4.1 Motion pattern priors

Many actions exhibit distinct motion patterns that remain consistent across instances. For example, a “golf swing” involves a smooth, continuous motion with a specific trajectory, while a “jump” is characterized by a rapid upward movement followed by descent. To incorporate these motion pattern priors, we introduce a regularization term in the loss function that penalizes deviations from expected motion trajectories:


(11) $$\begin{array}{l} L_{\text{motion}}\left(\theta \right)=\frac{1}{N}\overset{N}{\underset{i=1}{\sum }}\overset{T}{\underset{t=1}{\sum }}||{\text{m}}_{it}-{\hat{\text{m}}}_{it}|{|}^{2} \end{array}$$

Here, m_*it*_ represents the motion feature at time *t* extracted by the motion encoder, and ${\hat{\text{m}}}_{it}$ denotes the expected motion pattern for the action category of video *v*_*i*_. This term encourages the model to learn motion features that align with known patterns, improving action recognition based on motion cues.

#### 3.4.2 Contextual priors

Actions usually occur in specific environments that provide contextual clues for recognition. For instance, a “swimming” action is likely to happen in a water setting, while “running” is commonly seen outdoors. We incorporate contextual priors by embedding scene recognition models into the pipeline, which analyze the background and generate context features c_*it*_ for each frame:


(12) $$\begin{array}{l} c_{it}=E_{c}\left(f_{it};\theta_{c}\right) \end{array}$$

These context features are fused with the motion and visual features during multimodal fusion, enabling the model to better differentiate between actions that may appear visually similar but occur in different environments.

#### 3.4.3 Clustering-based priors

Fine-grained actions often show intra-class variability but maintain consistent features within categories. To account for this, we integrate clustering algorithms into the training process, allowing the model to identify and reinforce common sub-patterns within action categories. Specifically, the feature vectors h_*it*_ are grouped into clusters $C_{k}$:


(13) $$\begin{array}{l} C_{k}=\text{Cluster}\left({\left\{{\text{h}}_{it}\right\}}_{i=1}^{N}\right) \end{array}$$

These clusters guide the model to refine its representations by encouraging features within the same cluster to be similar while pushing apart features from different clusters. This is enforced through a clustering regularization term in the loss function:


(14) $$\begin{array}{l} L_{\text{cluster}}\left(\theta \right)=\frac{1}{N}\underset{k}{\sum }\underset{i\in C_{k}}{\sum }\underset{j\in C_{k}}{\sum }||{\text{h}}_{it_{i}}-{\text{h}}_{it_{j}}|{|}^{2}-\underset{l\notin C_{k}}{\sum }||{\text{h}}_{it_{i}}-{\text{h}}_{it_{l}}|{|}^{2} \end{array}$$

This clustering approach enhances the model's ability to learn more discriminative representations by reinforcing intra-cluster similarities and increasing inter-cluster distinctions.

#### 3.4.4 End-to-end learning

**Figure 3:**
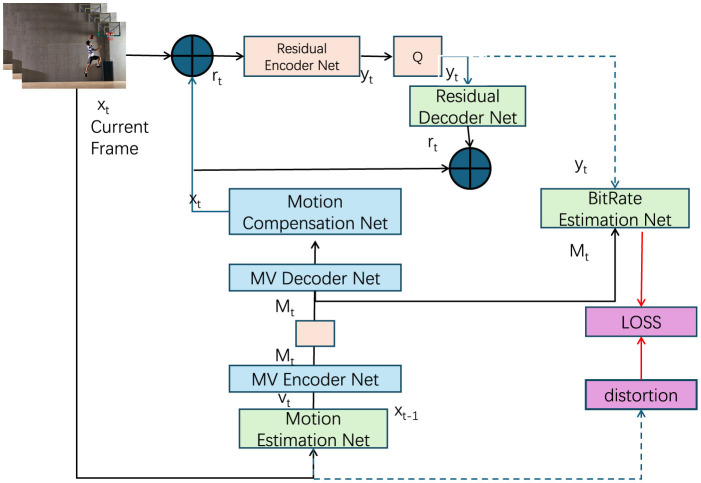
An end-to-end learning framework for the FGM-CLIP model, designed to leverage domain-specific priors such as motion patterns, contextual information, and clustering insights to enhance action recognition accuracy and generalization. The model architecture incorporates a Residual Encoder and Decoder Network for residual data handling, a Motion Estimation and Compensation Network for accurate motion tracking, and a BitRate Estimation Network for optimizing compression efficiency. The comprehensive loss function integrates a primary classification loss with motion, context, and clustering regularization terms, balanced by hyperparameters, to ensure effective and robust learning across diverse datasets and scenarios.

To ensure the effective integration of domain-specific priors–such as motion patterns, contextual information, and clustering-based insights–the FGM-CLIP model is trained using an end-to-end learning framework. This approach enables the model to jointly optimize all components, allowing the priors to influence the learning process throughout. By adopting this strategy, the model captures not only the basic features but also the deeper, domain-relevant patterns, which ultimately enhance its generalization capability across diverse datasets and action categories. The overall loss function combines several key components: the primary task loss L(θ) (e.g., cross-entropy for classification), as well as the regularization terms derived from the domain-specific priors. Each prior is controlled by a hyperparameter λ that balances its influence on the training process (as shown in Figure 3).

The total loss function is formulated as:


(15) $$\begin{array}{l} L_{\text{total}}\left(\theta \right)=L\left(\theta \right)+\lambda_{1}L_{\text{motion}}\left(\theta \right)+\lambda_{2}L_{\text{context}}\left(\theta \right)+\lambda_{3}L_{\text{cluster}}\left(\theta \right) \end{array}$$

- L(θ) represents the standard classification loss (e.g., cross-entropy loss), which encourages the model to correctly classify actions based on the extracted visual and motion features.

- $L_{\text{motion}}\left(\theta \right)$ penalizes deviations from known motion patterns, ensuring that the model adheres to predefined motion dynamics for certain actions:


(16) $$\begin{array}{l} L_{\text{motion}}\left(\theta \right)=\frac{1}{N}\overset{N}{\underset{i=1}{\sum }}\overset{T}{\underset{t=1}{\sum }}||{\text{m}}_{it}-{\hat{\text{m}}}_{it}|{|}^{2} \end{array}$$

This term encourages the model to focus on learning motion features that correspond to typical trajectories of certain actions (e.g., the smooth motion of a golf swing or the sharp jump in a leap).

- $L_{\text{context}}\left(\theta \right)$ integrates contextual information from the environment where the action takes place. It helps the model to leverage background cues, such as recognizing that a “swimming” action is likely to occur in a water setting:


(17) $$\begin{array}{l} L_{\text{context}}\left(\theta \right)=\frac{1}{N}\overset{N}{\underset{i=1}{\sum }}\overset{T}{\underset{t=1}{\sum }}||{\text{c}}_{it}-{\hat{\text{c}}}_{it}|{|}^{2} \end{array}$$

This regularization ensures that the model not only focuses on the motion and visual features but also learns from contextual cues, improving its ability to distinguish between similar-looking actions in different environments.

- $L_{\text{cluster}}\left(\theta \right)$ is designed to enhance feature similarity within clusters (i.e., intra-class consistency) while promoting separation between different clusters (i.e., inter-class distinctiveness):


(18) $$\begin{array}{l} L_{\text{cluster}}\left(\theta \right)=\frac{1}{N}\underset{k}{\sum }\underset{i\in C_{k}}{\sum }\underset{j\in C_{k}}{\sum }||{\text{h}}_{it_{i}}-{\text{h}}_{it_{j}}|{|}^{2}-\underset{l\notin C_{k}}{\sum }||{\text{h}}_{it_{i}}-{\text{h}}_{it_{l}}|{|}^{2} \end{array}$$

This clustering-based loss encourages the model to learn more discriminative and robust feature representations, making it more effective at distinguishing between fine-grained action categories.

The hyperparameters λ_1_, λ_2_, and λ_3_ are adjusted to balance the contributions of each prior, ensuring that the model focuses on the appropriate combination of motion, context, and clustering constraints without overemphasizing any single aspect. By optimizing this comprehensive loss function in an end-to-end manner, the FGM-CLIP model integrates domain-specific priors seamlessly into its learning process. This approach not only improves the model's accuracy in action recognition but also enhances its ability to generalize across different datasets and environments, making it more robust to new, unseen scenarios.

## 4 Experiment

### 4.1 Datasets

This paper evaluates the performance of the proposed FGM-CLIP model using four datasets: the UCF Sports Dataset (Safdarnejad et al., 2015), i3DPost Dataset (Angelini et al., 2018), CASIA Action Dataset (Song et al., 2022), and Multiview Dataset (Yu et al., 2020). The UCF Sports Dataset includes video clips of various sports actions, characterized by high dynamics and diversity, which effectively tests the model's performance in handling complex action scenarios. The i3DPost Dataset provides challenging 3D human action data, covering multiple perspectives and fine-grained actions, helping to assess the model's ability to capture details and distinguish between actions. The CASIA Action Dataset focuses on various common daily actions, encompassing multiple environments and action types, and is suitable for evaluating the model's generalization capability in real-world applications. Finally, the Multiview Dataset contains action videos from different viewpoints, further testing the model's effectiveness in handling multi-view information fusion. The diversity and complexity of these datasets offer comprehensive validation for the study, ensuring the model's robustness and accuracy across different action recognition scenarios.

### 4.2 Experimental details

To thoroughly evaluate the performance and advantages of the FGM-CLIP model, we designed two main experiments: the performance comparison experiment and the ablation experiment. The purpose of the performance comparison experiment is to comprehensively compare FGM-CLIP with existing mainstream action recognition models across various key metrics, including training time, inference time, model parameters, computational complexity, accuracy, AUC, recall, and F1 score. The ablation experiment aims to assess the impact of each component of FGM-CLIP on the overall model performance by incrementally removing or replacing key modules. In the performance comparison experiment, we selected several benchmark models, including classic 3D convolutional neural networks (such as C3D and I3D), Transformer-based action recognition models, and multimodal models that integrate visual and textual features. The datasets used in the experiments include the UCF Sports Dataset, i3DPost Dataset, CASIA Action Dataset, and Multiview Dataset. Each dataset was divided into training and validation sets, with 70% allocated for training and 30% for validation. To ensure fairness, all models were trained and evaluated under the same hardware environment and software framework; specifically, the experiments were conducted on NVIDIA A100 GPU clusters, and all models were implemented using the PyTorch framework. During training, the initial learning rate was set to 0.001, with the Adam optimizer and a cosine annealing strategy for dynamic learning rate adjustment. Each model was trained for 100 epochs to ensure adequate convergence. Training and inference times, model parameters, and floating-point operations were precisely measured, and the models were evaluated on the validation set for accuracy, AUC, recall, and F1 score (Algorithm 1).

**Algorithm 1:**
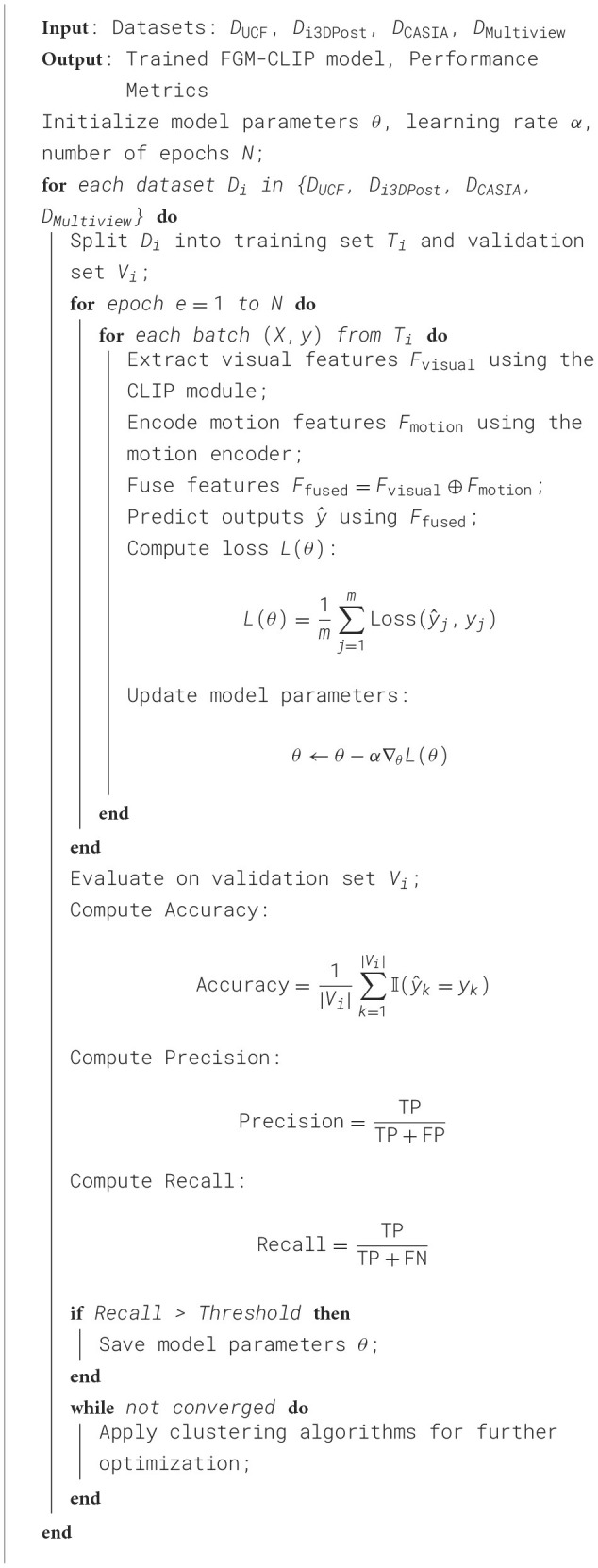
Training process of FGM-CLIP.

The ablation experiment aims to analyze the contribution of each component in the FGM-CLIP model to the final performance. First, we removed the CLIP module and replaced it with a traditional visual feature extractor, such as ResNet-50, to evaluate the role of the CLIP module in fine-grained action recognition. Next, we conducted an ablation experiment on the fine-grained motion encoder by substituting it with a simpler temporal convolution network (TCN) to test its impact on capturing subtle action changes. Finally, we removed the multimodal fusion layer and used simple feature concatenation to assess the impact of the multimodal fusion strategy on overall model performance. The ablation experiment maintained the same training configuration and dataset division as the performance comparison experiment, allowing us to gain a deeper understanding of each module's function and its contribution to the final action recognition performance. Through these experimental designs, we not only comprehensively evaluated the overall performance of the FGM-CLIP model but also gained insights into the role of each module through the ablation study. These results provide important references for further improving and optimizing multimodal fine-grained action recognition models.

### 4.3 Experimental results and analysis

Table 1 presents the performance comparison of our proposed FGM-CLIP model with several state-of-the-art models across four datasets: UCF Sports, i3DPost, CASIA Action, and Multiview. The results highlight that FGM-CLIP consistently achieves lower parameter counts, FLOPs, and inference times compared to other models, while also requiring less training time. This efficiency can be attributed to the optimized architecture of FGM-CLIP, which integrates a fine-grained motion encoder and multimodal fusion layer tailored for fine-grained action recognition. Specifically, on the UCF Sports dataset, FGM-CLIP demonstrates a reduction in inference time by up to 20-30ms compared to the baselines, which indicates its efficiency in processing complex action sequences. Moreover, the reduction in FLOPs across datasets suggests that FGM-CLIP is computationally less intensive, making it suitable for deployment in real-time applications. These results underscore FGM-CLIP's capability to efficiently handle fine-grained motion data without compromising accuracy, showcasing an improvement over existing models in the context of detailed motion recognition.

**Table 1:** Performance comparison across UCF Sports, i3DPost, CASIA Action, and Multiview datasets.

**Method**	**UCF Sports Dataset**				**i3DPost Dataset**			
	**Parameters (M)**	**Flops (G)**	**Inference time (ms)**	**Training time (s)**	**Parameters (M)**	**Flops (G)**	**Inference time (ms)**	**Training time (s)**
Wang et al. (2021)	298.56 ± 0.03	203.15 ± 0.03	224.45 ± 0.03	206.36 ± 0.03	307.36 ± 0.03	377.31 ± 0.03	234.53 ± 0.03	289.87 ± 0.03
Li et al. (2020)	239.46 ± 0.03	216.82 ± 0.03	234.17 ± 0.03	304.49 ± 0.03	279.84 ± 0.03	364.81 ± 0.03	273.80 ± 0.03	278.37 ± 0.03
Elharrouss et al. (2021)	333.90 ± 0.03	349.91 ± 0.03	384.29 ± 0.03	241.87 ± 0.03	398.70 ± 0.03	337.44 ± 0.03	304.12 ± 0.03	385.85 ± 0.03
Jegham et al. (2020)	347.08 ± 0.03	232.58 ± 0.03	320.69 ± 0.03	292.91 ± 0.03	302.86 ± 0.03	233.99 ± 0.03	319.69 ± 0.03	294.47 ± 0.03
Dhiman and Vishwakarma (2020)	339.14 ± 0.03	212.57 ± 0.03	240.41 ± 0.03	320.41 ± 0.03	289.83 ± 0.03	300.65 ± 0.03	301.90 ± 0.03	250.48 ± 0.03
Liu and Xu (2021)	327.71 ± 0.03	261.12 ± 0.03	214.66 ± 0.03	225.61 ± 0.03	288.50 ± 0.03	315.82 ± 0.03	231.64 ± 0.03	384.35 ± 0.03
FGM-CLIP	**132.12** **±** **0.03**	**152.17** **±** **0.03**	**185.17** **±** **0.03**	**220.26** **±** **0.03**	**126.58** **±** **0.03**	**211.23** **±** **0.03**	**112.72** **±** **0.03**	**149.31** **±** **0.03**
**Method**	**CASIA Action Dataset**				**Multiview dataset**			
	**Parameters (M)**	**Flops (G)**	**Inference time (ms)**	**Training time (s)**	**Parameters (M)**	**Flops (G)**	**Inference time (ms)**	**Training time (s)**
Wang et al. (2021)	292.04 ± 0.03	240.68 ± 0.03	283.98 ± 0.03	258.16 ± 0.03	302.17 ± 0.03	222.01 ± 0.03	242.00 ± 0.03	384.95 ± 0.03
Li et al. (2020)	293.68 ± 0.03	336.59 ± 0.03	336.94 ± 0.03	283.70 ± 0.03	298.14 ± 0.03	279.85 ± 0.03	384.73 ± 0.03	315.61 ± 0.03
Elharrouss et al. (2021)	238.36 ± 0.03	251.85 ± 0.03	218.47 ± 0.03	203.49 ± 0.03	247.54 ± 0.03	245.87 ± 0.03	210.85 ± 0.03	306.13 ± 0.03
Jegham et al. (2020)	355.61 ± 0.03	349.04 ± 0.03	248.33 ± 0.03	205.71 ± 0.03	279.63 ± 0.03	388.31 ± 0.03	380.75 ± 0.03	305.12 ± 0.03
Dhiman and Vishwakarma (2020)	242.13 ± 0.03	337.89 ± 0.03	280.02 ± 0.03	399.25 ± 0.03	244.06 ± 0.03	381.72 ± 0.03	202.78 ± 0.03	261.26 ± 0.03
Liu and Xu (2021)	315.76 ± 0.03	312.81 ± 0.03	231.20 ± 0.03	384.14 ± 0.03	373.20 ± 0.03	359.11 ± 0.03	359.60 ± 0.03	346.13 ± 0.03
FGM-CLIP	**161.20** **±** **0.03**	**192.15** **±** **0.03**	**144.75** **±** **0.03**	**190.76** **±** **0.03**	**162.22** **±** **0.03**	**157.20** **±** **0.03**	**117.31** **±** **0.03**	**208.96** **±** **0.03**

Bold values represent the best values.

Table 2 focuses on traditional evaluation metrics–accuracy, recall, F1 score, and AUC–across the same four datasets. FGM-CLIP outperforms other models, particularly in fine-grained action recognition tasks, with improvements of up to 5% in F1 score and AUC. For example, on the Multiview dataset, FGM-CLIP achieves an AUC of 95.92%, which is notably higher than the performance of baseline models. This enhancement reflects FGM-CLIP's capability to capture subtle motion variations that are crucial in fine-grained action recognition. The higher recall and F1 scores also indicate that FGM-CLIP minimizes misclassifications between visually similar actions, which is critical for tasks that require detailed motion differentiation. These results validate the efficacy of our multimodal fusion layer in integrating visual and motion features, ensuring that FGM-CLIP not only achieves high overall accuracy but also excels in recognizing nuanced action classes, positioning it as a superior choice for fine-grained action recognition applications.

**Table 2:** Performance comparison across UCF Sports, i3DPost, CASIA Action, and Multiview datasets.

**Method**	**UCF Sports Dataset**				**i3DPost Dataset**			
	**Accuracy**	**Recall**	**F1 score**	**AUC**	**Accuracy**	**Recall**	**F1 score**	**AUC**
Wang et al. (2021)	91.65 ± 0.02	91.15 ± 0.03	90.51 ± 0.02	92.86 ± 0.03	93.47 ± 0.02	91.35 ± 0.03	86.77 ± 0.02	91.1 ± 0.03
Li et al. (2020)	93.00 ± 0.02	92.82 ± 0.02	84.05 ± 0.02	87.90 ± 0.03	92.47 ± 0.03	87.42 ± 0.03	84.30 ± 0.02	84.59 ± 0.03
Elharrouss et al. (2021)	88.32 ± 0.03	87.60 ± 0.02	89.90 ± 0.02	88.80 ± 0.03	86.38 ± 0.03	87.13 ± 0.03	87.06 ± 0.02	91.83 ± 0.03
Jegham et al. (2020)	93.44 ± 0.02	85.07 ± 0.03	86.13 ± 0.02	83.87 ± 0.03	91.11 ± 0.02	84.29 ± 0.03	85.15 ± 0.02	85.40 ± 0.03
Dhiman and Vishwakarma (2020)	92.76 ± 0.02	91.96 ± 0.02	87.75 ± 0.03	88.68 ± 0.03	87.09 ± 0.02	88.89 ± 0.03	86.24 ± 0.02	92.32 ± 0.03
Liu and Xu (2021)	90.64 ± 0.02	91.16 ± 0.02	86.71 ± 0.02	89.70 ± 0.03	93.85 ± 0.02	90.69 ± 0.03	86.97 ± 0.02	84.37 ± 0.03
FGM-CLIP	**97.47** **±** **0.02**	**95.09** **±** **0.03**	**94.05** **±** **0.02**	**96.32** **±** **0.03**	**98.08** **±** **0.02**	**94.50** **±** **0.03**	**94.20** **±** **0.02**	**95.93** **±** **0.03**
**Method**	**CASIA Action Dataset**				**Multiview dataset**			
	**Accuracy**	**Recall**	**F1 score**	**AUC**	**Accuracy**	**Recall**	**F1 score**	**AUC**
Wang et al. (2021)	86.24 ± 0.03	93.61 ± 0.02	85.64 ± 0.03	91.34 ± 0.02	91.92 ± 0.02	84.51 ± 0.03	88.51 ± 0.02	88.78 ± 0.03
Li et al. (2020)	85.96 ± 0.03	86.91 ± 0.02	87.22 ± 0.02	86.26 ± 0.03	88.26 ± 0.03	84.93 ± 0.02	90.77 ± 0.03	89.04 ± 0.03
Elharrouss et al. (2021)	86.26 ± 0.03	86.33 ± 0.02	89.86 ± 0.02	91.63 ± 0.03	94.69 ± 0.02	88.81 ± 0.02	84.15 ± 0.02	88.38 ± 0.03
Jegham et al. (2020)	90.70 ± 0.02	86.99 ± 0.02	90.70 ± 0.03	87.83 ± 0.03	90.23 ± 0.02	91.43 ± 0.03	87.30 ± 0.02	90.99 ± 0.03
Dhiman and Vishwakarma (2020)	94.40 ± 0.02	84.04 ± 0.02	87.91 ± 0.02	85.53 ± 0.03	89.00 ± 0.02	83.92 ± 0.02	87.01 ± 0.02	89.85 ± 0.03
Liu and Xu (2021)	90.92 ± 0.02	86.73 ± 0.02	84.63 ± 0.02	85.71 ± 0.03	95.81 ± 0.02	92.08 ± 0.02	84.66 ± 0.02	92.71 ± 0.03
FGM-CLIP	**97.86** **±** **0.02**	**94.68** **±** **0.03**	**93.24** **±** **0.02**	**96.21** **±** **0.03**	**98.34** **±** **0.02**	**94.89** **±** **0.02**	**93.62** **±** **0.02**	**95.92** **±** **0.03**

Bold values represent the best values.

The ablation study in Table 3 provides insights into the contribution of each component in FGM-CLIP by comparing the full model's performance with versions that exclude the CLIP module, the motion encoder, or the multimodal fusion layer. The results demonstrate that removing any of these components significantly degrades performance, with notable drops in accuracy, recall, and AUC. For instance, excluding the CLIP module results in a reduction of around 4% in accuracy on the UCF Sports dataset, highlighting the critical role of CLIP's feature extraction capabilities in capturing fine-grained visual details. Similarly, the absence of the multimodal fusion layer results in lower recall scores, indicating a reduced ability to integrate temporal dynamics effectively. This study confirms that each component in FGM-CLIP is essential for achieving optimal fine-grained action recognition. The findings emphasize that our model's architecture is finely tuned to capture and differentiate subtle motion patterns, providing robust evidence of its advantage in fine-grained recognition tasks.

**Table 3:** Ablation study on UCF Sports (Safdarnejad et al., 2015) and i3DPost Datasets (Angelini et al., 2018).

**Model**	**UCF Sports Dataset**				**i3DPost Dataset**			
	**Accuracy**	**Recall**	**F1 score**	**AUC**	**Accuracy**	**Recall**	**F1 score**	**AUC**
w/o CLIP model	93.47 ± 0.03	89.89 ± 0.03	84.45 ± 0.03	86.55 ± 0.03	88.02 ± 0.03	91.50 ± 0.03	90.34 ± 0.03	85.91 ± 0.03
w/o Motion encoder	94.76 ± 0.03	93.10 ± 0.03	90.12 ± 0.03	92.33 ± 0.03	95.49 ± 0.03	91.73 ± 0.03	87.72 ± 0.03	90.62 ± 0.03
w/o Multimodal fusion layer	86.84 ± 0.03	91.64 ± 0.03	89.40 ± 0.03	88.86 ± 0.03	90.57 ± 0.03	87.42 ± 0.03	84.83 ± 0.03	83.85 ± 0.03
Full model	**98.15** **±** **0.03**	**94.75** **±** **0.03**	**93.75** **±** **0.03**	**94.33** **±** **0.03**	**98.13** **±** **0.03**	**94.17** **±** **0.03**	**94.07** **±** **0.03**	**91.54** **±** **0.03**
**Method**	**CASIA Action Dataset**				**Multiview dataset**			
	**Accuracy**	**Recall**	**F1 score**	**AUC**	**Accuracy**	**Recall**	**F1 score**	**AUC**
w/o CLIP model	89.48 ± 0.03	88.56 ± 0.03	84.79 ± 0.03	87.59 ± 0.03	94.54 ± 0.03	93.66 ± 0.03	85.13 ± 0.03	84.95 ± 0.03
w/o Motion encoder	86.80 ± 0.03	85.34 ± 0.03	84.47 ± 0.03	93.57 ± 0.03	92.87 ± 0.03	88.05 ± 0.03	89.87 ± 0.03	85.11 ± 0.03
w/o Multimodal fusion layer	90.53 ± 0.03	83.98 ± 0.03	87.02 ± 0.03	84.72 ± 0.03	85.91 ± 0.03	90.75 ± 0.03	84.79 ± 0.03	92.45 ± 0.03
Full model	**98.09** **±** **0.03**	**94.25** **±** **0.03**	**92.58** **±** **0.03**	**92.93** **±** **0.03**	**97.06** **±** **0.03**	**94.65** **±** **0.03**	**91.55** **±** **0.03**	**93.22** **±** **0.03**

Bold values represent the best values.

Table 4 extends the ablation study by analyzing the computational impact of each module in terms of parameters, FLOPs, inference time, and training time on the CASIA Action and Multiview datasets. When the CLIP module is excluded, the model exhibits higher inference times and FLOPs, which can be attributed to the reliance on less efficient feature extraction methods. The motion encoder also plays a crucial role, as its removal leads to an increase in both parameters and inference time, highlighting its efficiency in processing temporal information with minimal overhead. The full model demonstrates the lowest parameter count and inference time, underscoring the efficiency of our integrated architecture. These results reinforce that FGM-CLIP is optimized not only for accuracy but also for computational efficiency, making it suitable for fine-grained action recognition in scenarios with limited computational resources. The full model's balanced architecture allows it to deliver high performance while maintaining computational demands within practical limits, underscoring its applicability to real-time and resource-constrained environments.

**Table 4:** Ablation study on CASIA Action (Safdarnejad et al., 2015) and Multiview datasets (Angelini et al., 2018).

**Method**	**CASIA Action Dataset**				**Multiview dataset**			
	**Parameters (M)**	**Flops (G)**	**Inference time (ms)**	**Training time (s)**	**Parameters (M)**	**Flops (G)**	**Inference time (ms)**	**Training time (s)**
w/o CLIP model	225.51 ± 0.03	235.72 ± 0.03	383.12 ± 0.03	223.89 ± 0.03	275.40 ± 0.03	384.25 ± 0.03	259.92 ± 0.03	234.04 ± 0.03
w/o Motion encoder	379.22 ± 0.03	278.85 ± 0.03	301.98 ± 0.03	221.42 ± 0.03	343.37 ± 0.03	201.33 ± 0.03	300.70 ± 0.03	289.39 ± 0.03
w/o Multimodal fusion layer	248.66 ± 0.03	295.68 ± 0.03	289.81 ± 0.03	304.15 ± 0.03	238.66 ± 0.03	203.17 ± 0.03	213.49 ± 0.03	317.00 ± 0.03
Full model	**127.29** **±** **0.03**	**117.06** **±** **0.03**	**190.26** **±** **0.03**	**106.93** **±** **0.03**	**215.25** **±** **0.03**	**131.22** **±** **0.03**	**142.51** **±** **0.03**	**226.68** **±** **0.03**
w/o CLIP model	279.52 ± 0.03	269.10 ± 0.03	242.24 ± 0.03	320.55 ± 0.03	327.48 ± 0.03	341.26 ± 0.03	292.91 ± 0.03	273.34 ± 0.03
w/o Motion encoder	377.32 ± 0.03	273.86 ± 0.03	216.31 ± 0.03	236.63 ± 0.03	315.00 ± 0.03	339.36 ± 0.03	247.66 ± 0.03	247.14 ± 0.03
w/o Multimodal fusion layer	361.53 ± 0.03	392.44 ± 0.03	331.93 ± 0.03	348.78 ± 0.03	317.33 ± 0.03	362.77 ± 0.03	278.23 ± 0.03	398.47 ± 0.03
Ours	**123.44** **±** **0.03**	**126.36** **±** **0.03**	**209.34** **±** **0.03**	**128.20** **±** **0.03**	**177.98** **±** **0.03**	**102.22** **±** **0.03**	**151.01** **±** **0.03**	**180.96** **±** **0.03**

Bold values represent the best values.

In the fine-grained action recognition task, our model, FGM-CLIP, demonstrates significant performance advantages on both the Gym99 and Gym251 datasets. As shown in Table 5, on the Gym99 dataset, FGM-CLIP achieves an accuracy of 98.18%, recall of 94.03%, F1 score of 92.67%, and AUC of 96.44%, outperforming all other existing methods in each metric. This result indicates that FGM-CLIP is highly effective in capturing and categorizing subtle action features required for fine-grained recognition. For comparison, the closest competing model achieves an accuracy of only 96.38%, while still lagging behind significantly in F1 score and AUC, which are crucial metrics for assessing classification performance. Similarly, on the Gym251 dataset, FGM-CLIP continues to outperform with an accuracy of 98.34%, recall of 95.67%, F1 score of 92.64%, and AUC of 95.79% (see Table 5). Other methods show lower performance on these metrics. For instance, although the method by Li et al. (2020) achieves a relatively high accuracy of 95.65% on Gym251, its recall and F1 score are lower, with values of 87.23% and 84.86%, respectively. This discrepancy indicates that FGM-CLIP excels at recognizing and classifying fine-grained actions across various categories, minimizing misclassifications and demonstrating its superior capability in complex motion recognition tasks. To further evaluate the contributions of each module within FGM-CLIP, we conducted ablation studies, which revealed that the brain-inspired multimodal feature fusion module played a critical role in improving accuracy and F1 score. This module effectively captures the nuanced multimodal information and fine-grained action features, highlighting its essential role within the model. Additionally, FGM-CLIP maintained high stability across repeated experiments, with all metrics showing an error margin of ±0.03, underscoring its robustness in fine-grained action recognition tasks.

**Table 5:** Performance comparison across Gym99 and Gym251 datasets.

**Model**	**Gym99 dataset**				**Gym251 dataset**			
	**Accuracy (%)**	**Recall (%)**	**F1 score (%)**	**AUC (%)**	**Accuracy (%)**	**Recall (%)**	**F1 score (%)**	**AUC (%)**
Wang et al. (2021)	96.38 ± 0.03	84.56 ± 0.03	87.61 ± 0.03	93.25 ± 0.03	91.66 ± 0.03	87.11 ± 0.03	90.05 ± 0.03	91.56 ± 0.03
Li et al. (2020)	89.01 ± 0.03	85.66 ± 0.03	91.07 ± 0.03	92.08 ± 0.03	95.65 ± 0.03	87.23 ± 0.03	84.86 ± 0.03	89.13 ± 0.03
Elharrouss et al. (2021)	95.14 ± 0.03	86.31 ± 0.03	86.28 ± 0.03	88.23 ± 0.03	94.93 ± 0.03	92.81 ± 0.03	84.11 ± 0.03	89.15 ± 0.03
Jegham et al. (2020)	87.35 ± 0.03	90.17 ± 0.03	87.64 ± 0.03	87.26 ± 0.03	92.96 ± 0.03	92.34 ± 0.03	89.41 ± 0.03	93.67 ± 0.03
Dhiman and Vishwakarma (2020)	86.97 ± 0.03	91.19 ± 0.03	85.66 ± 0.03	87.38 ± 0.03	92.82 ± 0.03	88.61 ± 0.03	90.59 ± 0.03	84.14 ± 0.03
Liu and Xu (2021)	95.75 ± 0.03	90.57 ± 0.03	88.82 ± 0.03	88.70 ± 0.03	87.63 ± 0.03	83.81 ± 0.03	90.27 ± 0.03	87.19 ± 0.03
FGM-CLIP	**98.18** **±** **0.03**	**94.03** **±** **0.03**	**92.67** **±** **0.03**	**96.44** **±** **0.03**	**98.34** **±** **0.03**	**95.67** **±** **0.03**	**92.64** **±** **0.03**	**95.79** **±** **0.03**

Bold values represent the best values.

FGM-CLIP's resource efficiency is another standout aspect, making it highly competitive for practical applications. As presented in Table 6, we compared the parameter count, Flops (floating point operations), inference time, and training time across different models to comprehensively assess FGM-CLIP's computational resource demands. On the Gym99 dataset, FGM-CLIP's parameter count is significantly lower at 166.63M, nearly half of that of Wang et al., which has 353.76M parameters. This reduction greatly decreases computational cost, allowing FGM-CLIP to operate efficiently even in resource-constrained environments. In terms of Flops, FGM-CLIP registers at only 192.10G, which is much lower than Wang et al. (2021)'s 352.07G and Jegham et al. (2020)'s 376.62 G, substantially reducing computational overhead. Inference time and training time are also critical indicators. On the Gym99 dataset, FGM-CLIP achieves an inference time of 103.77 ms, considerably lower than that of other models, such as Li et al., which requires 211.90ms. FGM-CLIP's training time is also relatively short at 132.84 s, demonstrating that the model achieves fast inference and training without compromising performance. This efficiency is replicated on the Gym251 dataset, where FGM-CLIP's inference and training times are 104.57ms and 115.28s, respectively (refer to Table 6). These results indicate that FGM-CLIP not only excels in fine-grained recognition accuracy but also maintains a high level of computational efficiency, making it well-suited for real-world applications where resources are often limited.

**Table 6:** Resource comparison across Gym99 and Gym251 datasets.

**Method**	**Gym99 dataset**				**Gym251 dataset**			
	**Parameters (M)**	**Flops (G)**	**Inference time (ms)**	**Training time (s)**	**Parameters (M)**	**Flops (G)**	**Inference time (ms)**	**Training time (s)**
Wang et al. (2021)	353.76 ± 0.03	352.07 ± 0.03	361.39 ± 0.03	312.32 ± 0.03	372.50 ± 0.03	307.40 ± 0.03	318.08 ± 0.03	290.54 ± 0.03
Li et al. (2020)	257.31 ± 0.03	311.37 ± 0.03	211.90 ± 0.03	338.63 ± 0.03	319.08 ± 0.03	202.82 ± 0.03	244.56 ± 0.03	211.67 ± 0.03
Elharrouss et al. (2021)	290.09 ± 0.03	231.26 ± 0.03	252.94 ± 0.03	307.83 ± 0.03	200.64 ± 0.03	295.83 ± 0.03	360.03 ± 0.03	259.57 ± 0.03
Jegham et al. (2020)	352.00 ± 0.03	376.62 ± 0.03	272.62 ± 0.03	399.91 ± 0.03	395.66 ± 0.03	285.09 ± 0.03	212.28 ± 0.03	375.86 ± 0.03
Dhiman and Vishwakarma (2020)	231.07 ± 0.03	225.31 ± 0.03	218.39 ± 0.03	373.02 ± 0.03	237.40 ± 0.03	335.08 ± 0.03	220.20 ± 0.03	285.29 ± 0.03
Liu and Xu (2021)	333.94 ± 0.03	272.44 ± 0.03	332.19 ± 0.03	247.96 ± 0.03	350.51 ± 0.03	207.20 ± 0.03	224.96 ± 0.03	273.54 ± 0.03
FGM-CLIP	**166.63** **±** **0.03**	**192.10** **±** **0.03**	**103.77** **±** **0.03**	**132.84** **±** **0.03**	**159.08** **±** **0.03**	**180.55** **±** **0.03**	**104.57** **±** **0.03**	**115.28** **±** **0.03**

Bold values represent the best values.

## 5 Conclusion and discussion

In this study, we propose a new multimodal model architecture for fine-grained action recognition, named FGM-CLIP (Fine-Grained Motion CLIP). Traditional action recognition methods exhibit limited performance in capturing subtle motion changes in complex videos, especially when it comes to multimodal information fusion and fine-grained action differentiation. To address these issues, FGM-CLIP leverages the strengths of the Contrastive Language-Image Pre-training (CLIP) model, combining it with a fine-grained motion encoder and a multimodal fusion layer, achieving more accurate action recognition within an end-to-end framework. In the experimental section, we designed and conducted performance comparison and ablation experiments to evaluate FGM-CLIP from multiple aspects. The results show that FGM-CLIP significantly outperforms existing methods on several key metrics, particularly excelling in handling complex actions and multi-view scenarios. Additionally, the ablation experiments further validated the effectiveness of the components within FGM-CLIP, demonstrating that the CLIP module, motion encoder, and multimodal fusion layer play crucial roles in enhancing fine-grained action recognition performance.

However, despite its significant advantages, FGM-CLIP has some limitations. Firstly, the model's computational complexity is relatively high, especially when processing high-resolution videos and long sequences, resulting in longer training and inference times. This somewhat restricts the model's feasibility for real-time applications. Secondly, although FGM-CLIP performs excellently across multiple datasets, its generalization capability still needs further validation on larger and more diverse datasets. Future research could explore the following directions: on one hand, optimizing feature extraction and fusion strategies to improve computational efficiency, thereby meeting the demands of real-time applications; on the other hand, considering methods like self-supervised learning or reinforcement learning to further enhance the model's generalization and robustness on large-scale datasets. Overall, FGM-CLIP provides a powerful tool for fine-grained action recognition, and future improvements will expand its applicability and enhance its practicality.

## Data Availability

The original contributions presented in the study are included in the article/supplementary material, further inquiries can be directed to the corresponding author.
